# Accumulated dose on daily iCBCT for rectal cancer: Effects of inter‐fraction bowel cavity motion volume

**DOI:** 10.1002/acm2.70524

**Published:** 2026-02-27

**Authors:** Hongwei Zeng, Xiangyu E, Su Zeng, Yue Feng, Jingping Yu, Minghe Lv, Ruping Zhao

**Affiliations:** ^1^ Department of Radiotherapy Shuguang Hospital Affiliated to Shanghai University of Traditional Chinese Medicine Shanghai China; ^2^ Department of Radiotherapy Changzhou Cancer Hospital Changzhou China

**Keywords:** bowel cavity motion volume, cone beam CT, dose accumulation, radiotherapy toxicity, rectal cancer

## Abstract

**Background:**

To assess how inter‐fraction bowel cavity (BC) motion volume influences accumulated dose in rectal cancer radiotherapy and to translate these effects into a clinically interpretable risk construct for acute bowel toxicity.

**Methods:**

This retrospective cohort included 28 consecutive rectal cancer patients treated with 50 Gy in 25 fractions on a Halcyon v3.0 linac with daily iterative cone beam CT (iCBCT). Patients with > 5% bladder‐volume or > 8% external‐contour variation across the course were excluded. For each fraction, per‐fraction dose was recalculated on the iCBCT using a scanner‐specific HU‐relative electron density calibration, rigidly aligned to the planning CT (pCT), deformably registered, and voxel‐wise accumulated on pCT. Dosimetric endpoints were PTV *D*_2%_, *D*_50%_, *D*_98%_ and whole‐bowel (WB) *D*_0.03cc_, *D*_150cc_. Associations between inter‐fraction BC motion volume change (ΔV_BC_) and accumulated dose difference (ΔD) were quantified using Spearman's f and ordinary‐least‐squares slopes (Gy per 10% BC expansion); same‐day effects were estimated with patient fixed‐effects models. To account for multiple testing, *p*‐values for the five primary endpoints were adjusted using the Benjamini–Hochberg false discovery rate (FDR). Clinical relevance was assessed with a published logistic normal‐tissue complication probability (NTCP) model based on WB *V*_45_, defined as the WB volume receiving ≥ 45 Gy.

**Results:**

Accumulated dose analyses showed selective elevation of high‐dose metrics with preservation of near‐minimum target coverage. Compared with the plan, accumulated PTV *D*_2%_ and *D*_50%_ increased by 0.87 Gy and 0.61 Gy, respectively, whereas PTV *D*_98%_ exhibited no meaningful change. WB *D*_0.03cc_ and *D*_150cc_ increased by 1.21 Gy and 1.18 Gy, respectively. Inter‐fraction BC expansion was strongly associated with high‐dose escalation: for each 10% increase in BC volume, accumulated WB *D*_0.03cc_ increased by 0.31 Gy (95% CI 0.18–0.44; ρ = 0.78) and PTV *D*_2%_ by 0.29 Gy (0.15–0.37; ρ = 0.66). After FDR adjustment, no endpoint met *p* < 0.05, but the strongest associations (PTV *D*_2%_, WB *D*_0.03cc_) remained near‐significant and directionally consistent with large, biologically plausible effect sizes. Application of the WB *V*_45_ NTCP model indicated modest but consistent risk increments after accumulation, with NTCP for ≥ grade 2 toxicity increasing from 0.75 to 0.79 (+0.04; *p* = 0.001) and NTCP for grade 3 toxicity increasing from 0.80 to 0.84 (+0.04; *p *= 0.002).

**Conclusion:**

Daily iCBCT‐based dose accumulation identifies inter‐fraction BC motion volume as a selective driver of high‐dose escalation to the PTV and WB while largely preserving near‐minimum target coverage. Accumulated WB *V*_45_ may serve as a practical, delivery‐based marker for on‐treatment surveillance and risk‐adapted intervention, complementing plan‐based evaluation in rectal cancer radiotherapy.

## 1 INTRODUCTION

Pelvic radiotherapy for rectal cancer extends over several weeks, during which evolving pelvic anatomy can destabilize planned dose distributions and shift high‐dose regions toward radiosensitive bowel loops.^1^, ^2^, ^3^ The bowel cavity (BC), the intraluminal gas and fluid space, is a principal driver of this variability: peristalsis and fluctuating luminal contents alter its volume and distribution, thereby changing both bowel position and the electron‐density map used for dose calculation.^4^, ^5^ Although contemporary image guidance and prudent margins mitigate geometric uncertainties, they do not fully address cumulative day‐to‐day variation in luminal gas distribution and electron density.^6^, ^7^ Multiple lines of evidence from both cone beam computed tomography (CBCT)‐based dose accumulation and magnetic resonance imaging (MRI)‐guided adaptive workflows therefore converge on the conclusion that accurate evaluation of delivered dose is essential for capturing clinically meaningful gastrointestinal toxicity risk.^8^, ^9^

Despite this clinical imperative, course‐wide characterizations of BC‐driven dose effects remain limited. Many studies reconstruct only selected fractions or rely on relatively stable surrogates such as bladder or rectal metrics that may underrepresent the inter‐fraction bowel changes captured by BC motion volume. Recent CBCT and MRI guided analyses indicate that accumulated dose correlates more strongly with bowel or rectal toxicity than the planned dose.^10^, ^11^ Moreover, inter‐fraction anatomy studies demonstrate that factors closely linked to BC behavior, including intraluminal air and variable visceral filling, measurably perturb dose distributions and underscore the need to move beyond surrogate organs when explaining day‐to‐day fluctuations.^12^ Against this background, two areas warrant clarification. First, the magnitude and consistency with which inter‐fraction change in BC volume translates into cumulative dose differences in high‐dose target metrics and whole‐bowel (WB, including small and large bowel) endpoints across the course. Second, how these BC‐driven dosimetric differences map onto clinically interpretable risk constructs that support decision‐making and risk communication.

In this study, we performed dose accumulation across the full course on daily iterative CBCT (iCBCT) in a homogeneous cohort of patients receiving radiotherapy for rectal cancer. Per‐fraction doses were recalculated on each image set and mapped to the planning computed tomography (pCT) for voxel‐wise accumulation using deformable registration. The aim was to quantify how inter‐fraction change in BC motion volume relates to cumulative dose differences, expressed as effect sizes in Gy per ten percent increase in BC motion volume, and to assess clinical relevance by estimating normal tissue complication probability for acute bowel toxicity using established WB models.

## 2 METHODS AND MATERIALS

### 2.1 Patient data

We conducted a retrospective analysis of consecutively treated rectal cancer patients at Shuguang Hospital Affiliated to Shanghai University of Traditional Chinese Medicine (December 2022 to May 2024). Daily iCBCTs acquired throughout treatment were reviewed. To ensure anatomical stability, we excluded patients with > 5% bladder‐volume variation or > 8% change in external‐contour across the course. This selection was intended to minimize confounding from bladder filling and body habitus changes that can dominate pelvic dose variation and introduce additional uncertainty in dose mapping, thereby improving internal validity for isolating the dosimetric impact of BC motion volume. No standardized dietary protocol was mandated during radiotherapy to minimize BC volume, and no pharmacologic bowel preparation was used for this purpose. Twenty‐eight patients met eligibility (median age 69 years, range 48–86 years). Baseline anatomical parameters were summarized in Table 1. The pCT was acquired on a SOMATOM Confidence 20 scanner (Siemens, Berlin, Germany) in the supine position, 5 mm slice thickness. Treatments were delivered on a Halcyon v3.0 (Varian, CA, USA) with 6 MV FFF photons to 50 Gy in 25 fractions to the PTV using a 12‐field coplanar intensity modulated radiation therapy (IMRT) technique. Treatment plans were generated in Eclipse v16.1 (Varian, CA, USA) using the analytical anisotropic algorithm (AAA) for dose calculation. Before each fraction, pelvic‐protocol iCBCT was obtained (120 kV, 1080 mAs), yielding 700 datasets.

**TABLE 1:** Patient anatomical parameters (*n* = 28).

	Mean (range)	Inter‐fraction deviation mean (range)
PTV volume (cc)	1170 (876–1532)	N/A
Bladder volume (cc)	246 (186–270)	3.22% (1.08%–4.79%)
External‐contour volume (cc)	12618 (11290–14937)	4.43% (0.08%–7.13%)

### 2.2 HU‐to‐density calibration for iCBCT dose recalculation

To enable daily dose recalculation on iCBCT, we derived a scanner‐specific HU‐to‐relative electron density (RED) calibration using an independent cohort of 106 pelvic patients with paired iCBCT‐pCT acquired within 30 min (median: 18 min) under identical immobilization, thereby minimizing anatomical and setup variability. For each patient, a reference 50 Gy in 25 fractions IMRT plan was created on the pCT and rigidly registered to the paired iCBCT using pelvic bony landmarks with six degrees of freedom. Dose recalculation was then performed on the iCBCT based on this alignment.

**FIGURE 1:**
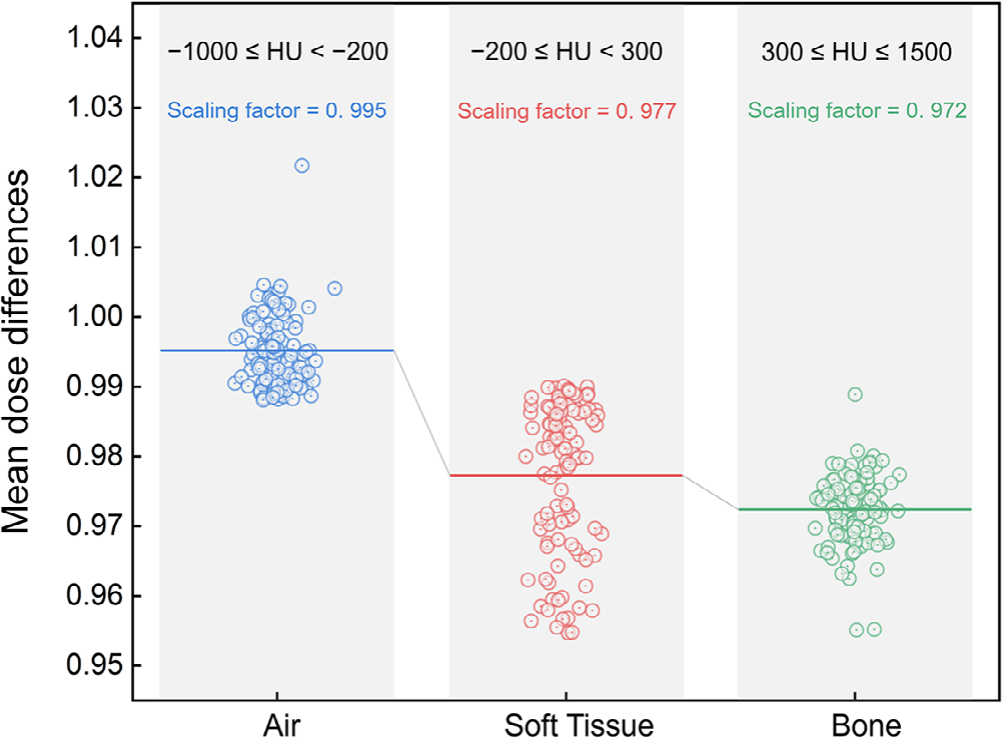
Scanner‐specific HU–RED curve for iCBCT‐based daily dose recalculation on the halcyon system. The curve was derived from 106 paired pCT‐iCBCT datasets using a three‐segment fit across air (−1000 ≤ HU < −200), soft tissue (−200 ≤ HU < 300), and bone (300 ≤ HU ≤ 1500) regions. The corresponding mean dose difference scaling factors for these regions were 0.995, 0.977, and 0.972, respectively. This calibration was subsequently implemented for iCBCT dose recalculation to ensure dosimetric consistency with pCT‐based planning.

As illustrated in Figure 1, a three‐segment HU‐RED curve was constructed based on the mean dose differences observed in three tissue‐equivalent HU ranges: air (−1000 ≤ HU < −200), soft tissue (−200 ≤ HU < 300), and bone (300 ≤ HU ≤ 1500). The corresponding mean dose difference scaling factors for these regions were 0.995, 0.977, and 0.972, respectively, and were used to parameterize the piecewise linear calibration. Calibration accuracy was evaluated in an independent 20‐patient validation subset. Agreement with pCT was demonstrated by a mean absolute HU error of 21 ± 5 HU and a gamma‐pass rate of 95.2 ± 1.8% using 2%/2 mm criteria, indicating stable dosimetric consistency. The resulting HU‐RED table was implemented in Eclipse v16.1 for daily iCBCT dose recalculation using the AAA. The same dose calculation algorithm was applied to both pCT and iCBCT to ensure algorithmic consistency. The standard Halcyon treatment couch was included in dose calculations for both pCT and iCBCT, and no additional patient‐specific scatter corrections or intervention‐dependent adjustments were applied.

### 2.3 BC segmentation

The BC was defined as intraluminal gas within small and large bowel, excluding stomach and rectum. Automatic segmentation of the small and large bowel on each iCBCT was initially performed using AccuContour v3.1 (Manteia, Xiamen, China) and subsequently reviewed by a senior radiation oncologist with more than 10 years of experience in pelvic radiotherapy. Manual editing was then applied to exclude the bowel wall and non‐gaseous intraluminal contents, yielding the final BC contours. To prioritize regions most relevant to target coverage, the BC boundaries were limited to a fixed extent of 5 mm above and below the PTV. In this cohort, the mean HU value of the final BC was −902.3 ± 38.8 HU, which is theoretically consistent with that of intraluminal gas.

### 2.4 Deformable registration and dose accumulation

**FIGURE 2:**
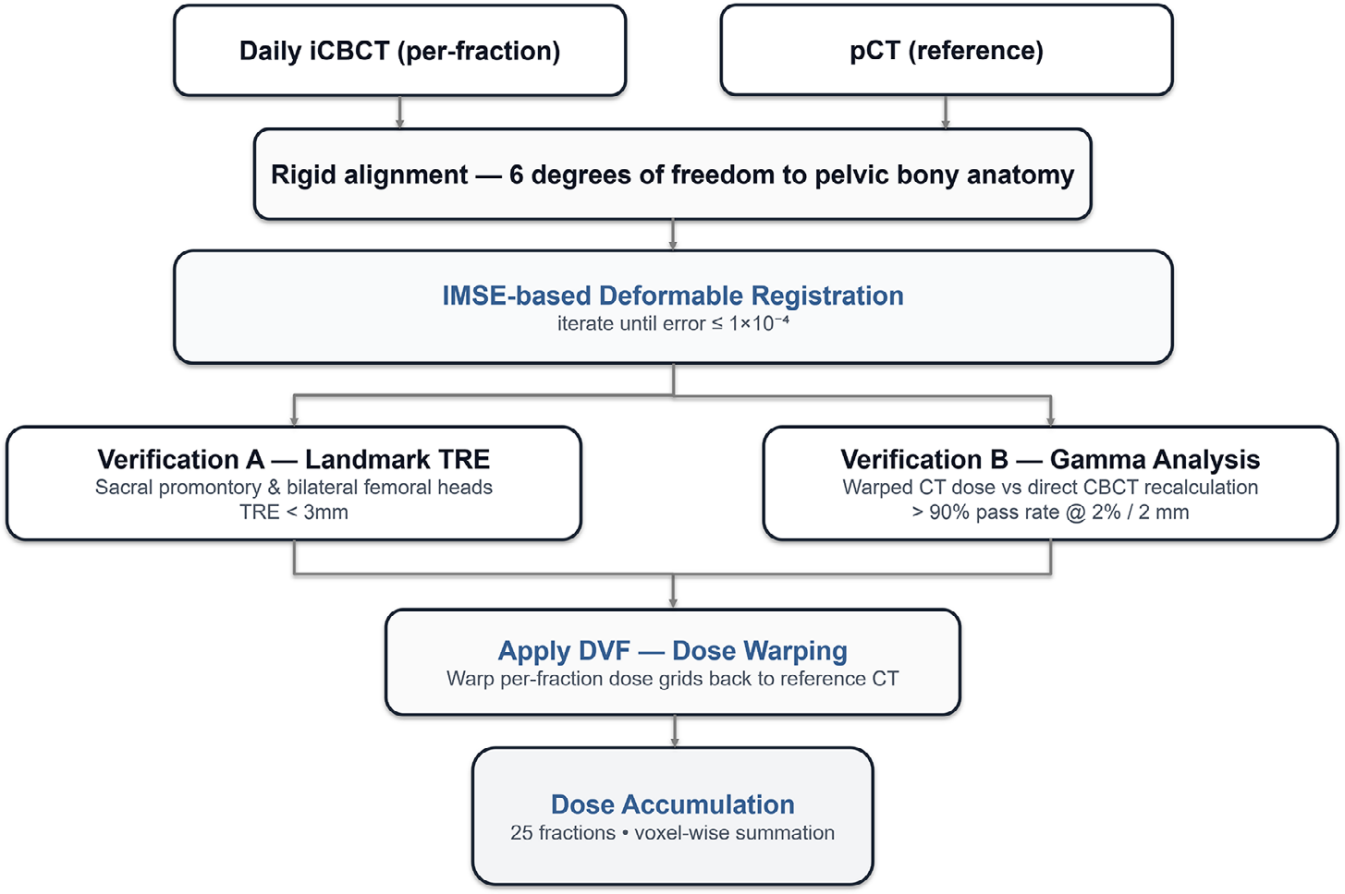
Workflow of dose accumulation. For each fraction, dose was recalculated on the iCBCT, rigidly aligned to the pCT, deformably registered using the IMSE‐based framework to obtain a DVF, and then warped back to the pCT for voxel‐wise summation across all 25 fractions to generate the accumulated dose distribution.

As shown in Figure 2, each fraction underwent rigid alignment to pCT (pelvic bones, six degrees of freedom) followed by deformable registration using the AccuLearning workstation v2.2 (Manteia, Xiamen, China) implementing the indescribable multi‐modal spatial evaluator (IMSE) framework.^13^ This self‐supervised algorithm learns a neural evaluator to quantify spatial discrepancies between multimodal images and drives an optimization network to generate the deformation vector field (DVF). The DVFs were iteratively refined until IMSE convergence, defined as an evaluator error ≤ 1×10^−^^4^. For each fraction, registrations were accepted only when convergence was achieved and quality assurance criteria were satisfied. Geometric accuracy was assessed on pelvic bony landmarks, requiring a landmark‐based target registration error (TRE) ≤ 3 mm at the sacral promontory and bilateral femoral heads. Dosimetric consistency was evaluated by gamma analysis within the pelvic region of interest (ROI), requiring gamma‐pass rates (2%/2 mm) ≥ 90% between the warped pCT dose and the directly recalculated iCBCT dose. In addition, intraluminal gas interfaces in regions of steep dose gradients were reviewed using ROI‐based overlay checks to ensure anatomically plausible deformations and to mitigate potential artifacts from air tissue discontinuities. If any criterion was not met, deformable registration was repeated with adjusted similarity ROI and regularization settings; when needed, extreme HU voxels were excluded from the similarity ROI to reduce the influence of air tissue interfaces. Fractions with persistent QA failure were to be excluded from dose accumulation according to protocol. In the final dataset, all 700 fractions (28 patients × 25 fractions) met the acceptance criteria and were retained. The validated DVFs were then used to propagate structures to each iCBCT and to warp per‐fraction doses back to the pCT, after which voxel‐wise summation across 25 fractions yielded accumulated dose distributions.

### 2.5 Dosimetric analysis

In this study, PTV endpoints included *D*_2%_ (Gy), *D*_50%_ (Gy), and *D*_98%_ (Gy), where *D*_2%_, *D*_50%_, and *D*_98%_ represent the doses received by 2%, 50%, and 98% of the PTV volume, respectively. WB endpoints included *D*_0.03cc_ (Gy) and *D*_150cc_ (Gy), where *D*_0.03cc_ and *D*_150cc_ represent the doses delivered to 0.03 cc and 150 cc of the WB volume, respectively. For each patient, metrics were extracted from the approved plan, per‐fraction delivered (inter‐fraction), and accumulated doses (sum over daily iCBCT recalculations). As indicated by Equations. 1 and 2, the primary endpoints were the absolute (Δ) and relative (δ) differences.

(1) $$\Delta D_{x}=D_{x,fraction/accum}-D_{x,plan}$$

(2) $$\delta D_{x}=\frac{D_{x,fraction/accum}-D_{x,plan}}{D_{x,plan}}\times 100\%$$

### 2.6 Quantifying inter‐fraction BC motion volume and statistical analysis

Inter‐fraction BC motion volume (ΔV_BC_) was defined as the relative change in BC volume at each treatment fraction compared with the volume at initial simulation. For patient‐level analyses, ΔV_BC_ was summarized as the median value across all 25 fractions. Associations between ΔV_BC_ and ΔD (PTV: *D*_2%_, *D*_50%_, *D*_98%_; WB: *D*_0.03cc_, *D*_150cc_) were tested using Spearman's ρ with bias‐corrected bootstrap 95% CIs (2,000 resamples). Accumulated level effects were estimated by ordinary least squares (OLS) regression (slope reported as Gy per 10% BC expansion; HC3 CIs). These OLS models quantify between‐patient associations between ΔV_BC_ and course‐wide ΔD. As a within‐patient validation, fixed‐effects (FE) linear models at the fraction level incorporated patient‐specific intercepts, a fraction‐index covariate, and slopes per 10% BC expansion with patient‐clustered robust SEs, thereby providing a within‐patient analysis that accounts for individual baselines and time trends and confirming that the observed associations are not attributable solely to between‐patient differences. Because several prespecified dosimetric endpoints were evaluated in parallel, *p*‐values from Spearman correlations and OLS regression slopes were additionally adjusted using the Benjamini–Hochberg false discovery rate (FDR) procedure. The five primary endpoints (PTV: *D*_2%_, *D*_50%_, *D*_98%_; WB: *D*_0.03cc_, *D*_150cc_) were treated as a single family for correction. Both raw and FDR‐adjusted *p*‐values are reported. FE model estimates were considered confirmatory analyses and were not included in the FDR correction.

### 2.7 Toxicity modeling

Acute radiation‐induced enteritis was estimated using a published WB logistic normal‐tissue complication probability (NTCP) model parameterized by *V*_45_ (cc), defined as the WB volume receiving ≥ 45 Gy, with *V*_50 _= 130 cc and *k* = 1.1.^14^ The model was (Equation. 3):

(3) $$NTCP=\frac{1}{1+{\left(\frac{V_{50}}{V_{45}}\right)}^{k}}$$

For each patient, NTCP ≥ grade 2 acute WB toxicity was computed separately from the planned dose volume histogram (DVH) and the accumulated DVH derived during treatment, using the RTOG acute gastrointestinal toxicity criteria,^15^ consistent with the endpoint of the original model. To appraise clinical validity, predicted NTCPs were compared against the observed incidence of acute enteritis in the study cohort.

### 2.8 Ethics approval and consent to participate

This study was conducted in accordance with the Declaration of Helsinki and was approved by Shuguang Hospital Affiliated to Shanghai University of Traditional Chinese Medicine. Written informed consent was obtained from all enrolled patients for the use of their imaging and clinical data for research purposes. All data were anonymized prior to analysis to ensure confidentiality.

## 3 RESULTS

For each patient (*n* = 28), inter‐fraction delivered‐planned deviations were computed and summarized by the within‐patient median across 25 fractions. As shown in Table 2, delivered doses were systematically higher than planned for high‐dose PTV metrics and for WB exposure. For the PTV, *D*_2%_ increased by 1.61 Gy (δ = 3.10%), with 16/28 patients (57.14%) exceeding |δ| ≥ 3%; *D*_50%_ increased by 1.18 Gy (δ = 2.37%), with 6/28 (21.43%) ≥ 3%, while *D*_98%_ remained close to plan. For the WB, *D*_0.03cc_ and *D*_150cc_ rose by 2.20 Gy (δ = 4.24%) and 2.01 Gy (δ = 3.96%), respectively; threshold analyses showed ≥ 1/2/3 Gy exceedances of 28/28 (100%), 18/28 (64.29%), 5/28 (17.86%) for *D*_0.03cc_ and 28/28 (100%), 15/28 (53.57%), 0/28 (0%) for *D*_150cc_. Collectively, delivery preferentially increased high‐dose exposure within the PTV (*D*_2%_, *D*_50%_) while preserving near‐minimum coverage (*D*_98%_) and produced clinically relevant upward shifts in WB hotspots and bulk dose.

**TABLE 2:** Median deviations between inter‐fraction delivered and planned dose for PTV and WB metrics across 28 patients, with proportions of patients exceeding predefined absolute (|Δ|, Gy) and relative (|δ|, %) thresholds.

	Median Δ (Gy) [95% CI]	Median δ (%) [95% CI]	Threshold	Exceeding threshold
PTV *D*_2%_	1.61 [1.43, 2.09]	3.10 [2.81, 4.00]	|δ| ≥ 3%	16/28 (57.14%)
PTV *D*_50%_	1.18 [1.02, 1.26]	2.37 [2.03, 2.51]	|δ| ≥ 3%	6/28 (21.43%)
PTV *D*_98%_	0.11 [−0.67, 0.21]	0.23 [−1.34, 0.44]	|δ| ≥ 3%	0/28 (0%)
WB *D*_0.03cc_	2.20 [1.92, 2.61]	4.24 [3.69, 5.03]	|Δ| ≥ 1 Gy	28/28 (100%)
			|Δ| ≥ 2 Gy	18/28 (64.29%)
			|Δ| ≥ 3 Gy	5/28 (17.86%)
WB *D*_150cc_	2.01 [1.79, 2.43]	3.96 [3.60, 4.78]	|Δ| ≥ 1 Gy	28/28 (100%)
			|Δ| ≥ 2 Gy	15/28 (53.57%)
			|Δ| ≥ 3 Gy	0/28 (0%)

**FIGURE 3:**
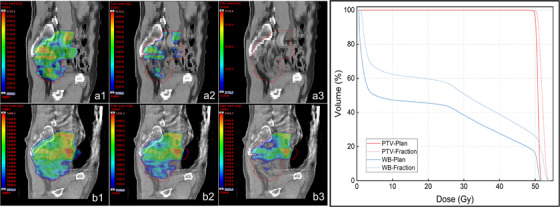
BC‐driven hotspot amplification in an illustrative case. Panels a1–a3 show the sagittal dose distribution of the original plan, illustrating the 50 Gy, 51 Gy, and near‐*D*max (51.79 Gy) isodose regions, respectively. Panels b1–b3 present the corresponding inter‐fraction recalculated dose distribution in the same view, showing the 50 Gy, 51 Gy, and 52 Gy isodose regions, with an observed *D*max of 54.08 Gy. The DVHs for both the PTV and WB show a rightward shift in the high‐dose tail, indicating increased upper‐dose exposure in the inter‐fraction plan.

Figure 3 illustrates an example case with marked inter‐fraction BC distension on iCBCT: the delivered *D*_max_ (maximum dose) increased from 51.79 Gy (plan) to 54.08 Gy (δ = 4.4%). On sagittal review, the 50–52 Gy isodose envelopes expanded almost exclusively within slices intersecting the dilated BC, whereas slices without distension showed minimal geometric deviation. DVHs exhibit a rightward shift confined to the high‐dose tail, while PTV *D*_98%_ remained near plan, illustrating the spatial selectivity underpinning the cohort‐level pattern.

After deformable accumulation across fractions, the fraction‐wise pattern persisted but with attenuated magnitudes. As shown in Table 3, PTV *D*_2%_ and *D*_50%_ remained higher than planned by 0.87 Gy (δ = 1.68%) and 0.61 Gy (δ = 1.23%), respectively; *D*_98%_ stayed near plan (Δ = 0.07 Gy; δ = 0.15%). For WB, *D*_0.03cc_ and *D*_150cc_ increased by 1.21 Gy and 1.18 Gy, respectively. Threshold analyses corroborated the clinical relevance of these modest but systematic shifts: for *D*_0.03cc_, 28/28 (100%), 20/28 (71.43%), and 1/28 (3.57%) patients were ≥ 0.5/1/2 Gy; for *D*_150cc_, the corresponding counts were 28/28 (100%), 27/28 (96.43%), and 0/28 (0%). These accumulated deviations were smaller than the inter‐fraction medians, indicating partial cross‐fraction compensation without full cancellation.

**TABLE 3:** Median deviations between accumulated and planned dose for PTV and WB metrics across 28 patients, with proportions of patients exceeding predefined absolute (|Δ|, Gy) and relative (|δ|, %) thresholds.

	Median Δ (Gy) [95% CI]	Median δ (%) [95% CI]	Threshold	Exceeding threshold
PTV *D*_2%_	0.87 [0.85, 1.07]	1.68 [1.66, 2.08]	|δ| ≥ 1%	28/28 (100%)
			|δ| ≥ 2%	11/28 (39.29%)
			|δ| ≥ 3%	0/28 (0%)
PTV *D*_50%_	0.61 [0.55, 0.74]	1.23 [1.10, 1.47]	|δ| ≥ 1%	20/28 (71.43%)
			|δ| ≥ 2%	6/28 (21.43%)
			|δ| ≥ 3%	0/28 (0%)
PTV *D*_98%_	0.07 [−0.44, 0.07]	0.15 [−0.88, 0.15]	|δ| ≥ 1%	15/28 (53.57%)
			|δ| ≥ 2%	5/28 (17.86%)
			|δ| ≥ 3%	0/28 (0%)
WB *D*_0.03cc_	1.21 [1.13, 1.39]	2.33 [2.18, 2.67]	|Δ| ≥ 0.5 Gy	28/28 (100%)
			|Δ| ≥ 1 Gy	20/28 (71.43%)
			|Δ| ≥ 2 Gy	1/28 (3.57%)
WB *D*_150cc_	1.18 [1.17, 1.34]	2.32 [2.26, 2.65]	|Δ| ≥ 0.5 Gy	28/28 (100%)
			|Δ| ≥ 1 Gy	27/28 (96.43%)
			|Δ| ≥ 2 Gy	0/28 (0%)

**FIGURE 4:**
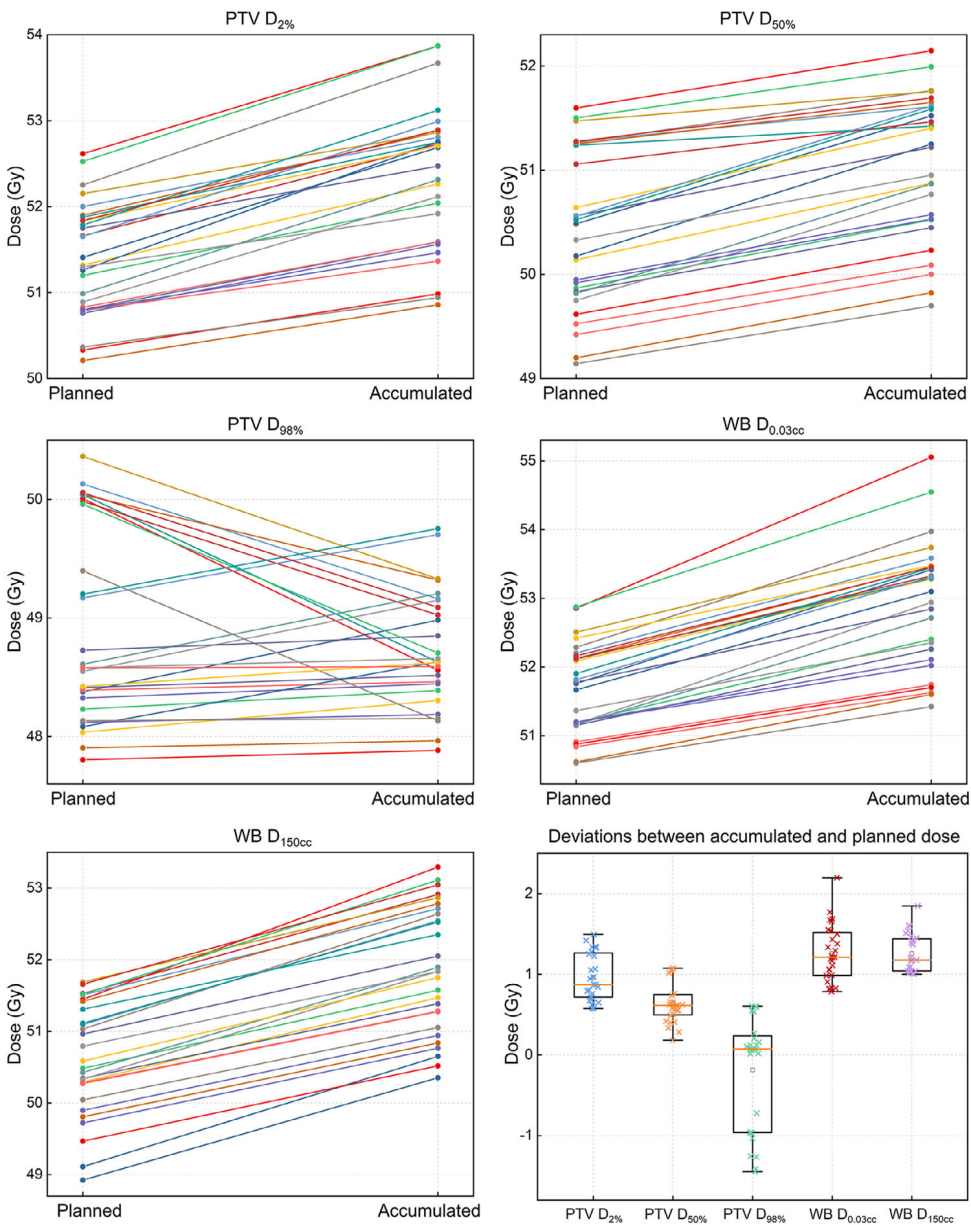
Panels show per‐patient paired values for PTV (*D*_2%_, *D*_50%_, *D*_98%_) and WB (*D*_0.03cc_, *D*_150cc_) endpoints, with each line connecting the planned to the accumulated dose. Accumulated high‐dose metrics (PTV *D*_2%_, *D*_50%_; WB *D*_0.03cc_, *D*_150cc_) shifted upward across the cohort, demonstrating systematic increases in upper‐dose exposure after daily recalculation and accumulation. In contrast, PTV *D*_98%_ remained tightly clustered around the prescription dose with near‐symmetric scatter, indicating minimal deviation. Companion boxplots summarized accumulated‐planned differences, highlighting positively shifted distributions for high‐dose metrics and negligible change for *D*_98%_.

Figure 4 illustrates the cohort‐level comparison of accumulated versus planned dose. High‐dose metrics (PTV *D*_2%_, *D*_50%_; WB *D*_0.03cc_, *D*_150cc_) showed a consistent upward tilt, indicating systematic increases with variable patient‐specific magnitudes. In contrast, PTV *D*_98%_ segments clustered tightly around 49 Gy with near symmetric scatter, reflecting negligible net change. Companion boxplots of accumulated‐planned differences corroborated these patterns, showing positively shifted distributions for the high‐dose PTV and WB metrics.

Associations between inter‐fraction ΔV_BC_ and accumulated dose differences were directionally consistent across endpoints, with the strongest and most precise effects in high‐dose metrics, as shown in Table 4. For WB *D*_0.03cc_, the accumulated‐level Spearman's ρ was 0.78 (95% CI 0.34–0.84); each 10% increase in BC volume corresponded to a 0.31 Gy increase (95% CI 0.18–0.44; *p *= 0.015) in OLS models and a same‐day FE slope of 0.39 Gy (95% CI 0.28–0.51; *p *= 0.002). For PTV *D*_2%_, ρ = 0.66 (95% CI 0.37–0.79) with an OLS slope of 0.29 Gy per 10% expansion (95% CI 0.15–0.37; *p *= 0.021) and an FE slope of 0.22 Gy (95% CI 0.14–0.31; *p *= 0.001). Medium or low dose metrics (PTV *D*_50%_, PTV *D*_98%_, WB *D*_150cc_) showed smaller slopes or wider CIs; notably, FE models detected modest but significant same‐day effects for PTV *D*_50%_ (0.09 Gy; 95% CI 0.03–0.17; *p *= 0.010) and WB *D*_150cc_ (0.15 Gy; 95% CI 0.07–0.20; *p *= 0.016). Overall, larger ΔV_BC_ was more consistently linked to increases in high‐dose PTV and WB exposure than to moderate or low dose endpoints.

**TABLE 4:** Association between inter‐fraction ΔV_BC_ and ΔD for each dosimetric endpoint. Spearman's ρ and OLS slopes are derived from accumulated‐level analyses, while FE slopes are from fraction‐level models adjusting for fraction index. Slopes are expressed in Gy per 10% BC expansion, with positive values indicating that greater ΔV_BC_ is associated with higher dose differences. Both raw and Benjamini–Hochberg FDR‐adjusted *p*‐values are reported for the five prespecified endpoints. Statistical significance was defined as FDR‐adjusted *p* < 0.05.

	ΔV_BC_ in accumulated‐level				ΔV_BC_ in fraction‐level	
	ρ [95% CI]	Slope [95% CI]	*p* (slope)	*p* (FDR‐adjusted)	FE slope [95% CI]	*p* (FE slope)
PTV *D*_2%_	0.66 [0.37, 0.79]	0.29 [0.15, 0.37]	0.021*	0.053	0.22 [0.14, 0.31]	0.001*
PTV *D*_50%_	0.32 [−0.05, 0.62]	0.14 [−0.16, 0.45]	0.352	0.440	0.09 [0.03, 0.17]	0.010*
PTV *D*_98%_	0.31 [−0.06, 0.61]	0.07 [−0.92, 1.01]	0.606	0.606	0.01 [−0.02, 0.03]	0.637
WB *D*_0.03cc_	0.78 [0.34, 0.84]	0.31 [0.18, 0.44]	0.015*	0.075	0.39 [0.28, 0.51]	0.002*
WB *D*_150cc_	0.21 [−0.16, 0.55]	0.10 [−0.17, 0.38]	0.156	0.260	0.15 [0.07, 0.20]	0.016*

After FDR adjustment, no endpoint met the *p* < 0.05 threshold. The dose‐anatomy relationships were therefore considered exploratory. Nevertheless, the strongest associations, particularly PTV *D*_2%_ and WB *D*_0.03cc_, remained near significant and directionally consistent. The effect sizes were large and biologically plausible, suggesting robust dose response patterns despite limited statistical power.

Applying the WB logistic NTCP model (≥ grade 2 acute WB toxicity) to planned and accumulated DVHs showed small but systematic increases with dose accumulation across toxicity strata. As shown in Table 5, grade 2 rose from 0.75 ± 0.05 to 0.79 ± 0.04 (*p *= 0.001); grade 3 increased from 0.80 ± 0.02 to 0.84 ± 0.02 (*p *= 0.002). WB *V*_45_ values demonstrated grade‐dependent clustering in this cohort. This retrospective pattern motivated exploratory triage bands centered around approximately 300 cc and 500 cc. Specifically, patients with *V*_45_ < 300 cc were predominantly grade 1 (mean 222.76 cc), those with 300–500 cc chiefly grade 2 (mean 423.61 cc), and those > 500 cc mainly grade 3 (mean 575.93 cc). In our cohort, acute WB toxicity occurred in 6/28 (21.43%) grade 1, 17/28 (60.71%) grade 2, and 5/28 (17.86%) grade 3 patients; thus, grade ≥ 2 toxicity was observed in 22/28 (78.57%). These acute WB toxicity data were available for the cohort and were used for qualitative comparison with modeled NTCP values. Although the study was not powered for formal predictive validation, the observed distribution of toxicity grades was consistent with the NTCP estimates derived from accumulated DVHs.

**TABLE 5:** Comparison of WB dose–Volume metrics and NTCP estimates between planned and accumulated doses stratified by toxicity grade. Values are presented as mean ± standard deviation. For all toxicity grades, accumulated dose metrics and NTCP values were significantly higher than those derived from planned doses (paired t‐test, *p* < 0.05).

Toxicity Grade	V_45_‐planned	V_45_‐accumulated	NTCP‐planned	NTCP‐accumulated	*p*
Grade 1 (6/28)	199.20 ± 46.14	222.76 ± 51.24	N/A	N/A	N/A
Grade 2 (17/28)	352.21 ± 66.13	423.61 ± 75.44	0.75 ± 0.05	0.79 ± 0.04	0.001*
Grade 3 (5/28)	460.82 ± 52.87	575.93 ± 58.66	0.80 ± 0.02	0.84 ± 0.02	0.002*

## 4 DISCUSSION

Across a homogeneous cohort treated with daily iCBCT recalculation and voxel‐wise accumulation, we delineated a coherent, biologically plausible chain linking inter‐fraction BC motion volume to delivered dose and modeled acute toxicity. Quantitatively, upper‐tail dose to both PTV and WB increased relative to plan (e.g., PTV *D*_2%_ by 0.87 Gy; WB *D*_0.03cc_ by 1.21 Gy; WB *D*_150cc_ by 1.18 Gy), whereas near‐minimum PTV coverage (*D*_98%_) was essentially preserved. Same‐day analyses indicated that each 10% expansion in BC volume was associated with roughly 0.31 Gy higher high‐dose endpoints. These contemporaneous effects only partially averaged out across the course, yielding an absolute increase of about 0.03 in NTCP for ≥ grade 2 acute WB toxicity. Accumulated DVHs also suggested pragmatic WB *V*_45_ demarcations near 300 cc and 500 cc that mirrored the observed grade distribution and provide interpretable bands for risk communication and decision‐making.^16^, ^17^ Importantly, these NTCP increments are model‐based estimates derived within the domain and assumptions of a published WB *V*_45_ logistic model. Accordingly, they should be interpreted as delivery‐informed risk indicators rather than definitive patient‐level toxicity predictions.

In the context of prior evidence, these observations both reaffirm and extend several established strands. Within pelvic radiotherapy for rectal cancer, multiple studies have identified bowel *V*_45_ as a practical correlate of acute gastrointestinal morbidity; our data reinforce this premise by anchoring *V*_45_‐based inference in delivered rather than planned exposure and by proposing clinically interpretable strata that align with the observed toxicity gradient.^18^ Systematic evaluations of pelvic organs at risk further indicate that high‐dose bowel volumes (e.g. *V*_40_–*V*_60_) track acute events, with loop‐based constraints such as *V*_45_ among the more discriminating descriptors.^19^ Our accumulation results converge with these dose‐volume‐outcome patterns while adding that daily BC‐driven perturbations preferentially inflate upper DVH tails even when target near‐minimum coverage is maintained. Beyond rectal cancer, delivered dose studies using CBCT or MR‐guided workflows have shown that dose of the course predicts toxicity more faithfully than baseline plan dosimetry.^20^, ^21^, ^22^ In particular, total or early fraction accumulated dose to hollow organs has outperformed plan metrics for forecasting acute symptom flares.^23^ Our findings align with this delivery versus plan gap and add dose resolved anatomical attribution by isolating BC variability as a proximal driver and quantifying its contribution at both fraction and course levels.

From a dose‐transport perspective, episodic BC expansion adjacent to steep isodose gradients can displace radiosensitive bowel loops into hotter envelopes and perturb pathlength and scatter in ways not fully compensated by fluence optimized under planning conditions.^24^, ^25^ These perturbations are spatially localized and time varying. Their dominant signature is a preferential rise in the upper DVH tail (PTV *D*_2%_, WB *D*_0.03cc_ and *D*_150cc_) with preservation of *D*_98%_, and this selective effect is reproduced by our same‐day slopes and persists, albeit attenuated, in the accumulated distribution. The observed effect sizes, on the order of a few tenths of a Gy per 10% BC expansion, are consistent with density sensitive behavior and provide a clinically interpretable scale for surveillance. The application of FDR correction improves statistical rigor but attenuates nominal significance. Even so, the strongest associations, particularly PTV *D*_2%_ and WB *D*_0.03cc_, remain directionally consistent with large and biologically plausible effect sizes. We therefore emphasize effect sizes and their uncertainty rather than making confirmatory claims of association.

However, even modest absolute escalations in upper‐tail dose can translate into non‐trivial increases in acute toxicity probability once integrated over clinically meaningful WB volumes. These findings motivate an exploratory on‐treatment surveillance concept in which accumulated WB *V*_45_ is tracked as a marker of delivered dose derived from daily iCBCT‐based dose recalculation. The approximate *V*_45_ bands around 300 cc and 500 cc are hypothesis‐generating triage ranges identified by retrospective clustering in this cohort and should not be interpreted as prospectively validated intervention thresholds. Operationally, automated BC segmentation combined with simple decision rules could provide a low‐overhead mechanism to flag patients for review. For example, a case may be flagged when accumulated WB *V*_45_ enters the 300–500 cc band with a sustained upward trajectory over successive fractions, or when WB *V*_45_ exceeds approximately 500 cc at any time. For flagged cases, a stepwise response can be considered, beginning with reinforcement of bowel preparation and dietary guidance per institutional practice and focused image review for marked distension or gas pockets near steep dose gradients, followed by symptom‐guided clinical assessment. If elevated WB *V*_45_ or hotspot metrics persist despite optimization of preparation and setup, the case may be prioritized for an adaptive review. Depending on local resources and availability, adaptive options may include selective replanning, repeat CT simulation, plan selection from a pre‐approved library, or online adaptation in centers where such workflows are implemented. Importantly, the stability criteria used in this study can be incorporated into broader pelvic workflows as a stratification rule. Anatomically stable patients with modest bladder‐volume and external‐contour variation are well suited for BC‐focused monitoring using accumulated *V*_45_ and hotspot metrics. In contrast, patients exceeding these stability thresholds may experience delivered‐dose perturbations dominated by more global anatomic deformation, in which case intensified review and broader adaptive strategies are likely more appropriate than directly applying the risk bands and effect estimates derived from an anatomically stable cohort. This framework is intended to illustrate how delivered‐dose information might be operationalized and requires prospective evaluation before being adopted as a clinical adaptive trigger.

This study has limitations. Firstly, this single‐center retrospective analysis used a fully specified, platform‐anchored workflow (scanner‐specific HU‐to‐density calibration and a defined deformable registration with quantitative quality control), which improves transparency and reproducibility. However, differences in imaging protocols, reconstruction kernels, and noise characteristics across vendors may affect HU stability and registration performance. Therefore, institutions adopting a similar workflow should perform system‐specific HU calibration and local validation of registration accuracy. Secondly, residual registration uncertainty in low contrast regions was unavoidable despite quantitative QA and may have led to local under or over estimation of dose. Thirdly, while AAA is widely used in clinical pelvic radiotherapy and supports internally consistent dose comparisons, its performance may be limited in gas‐containing bowel regions with strong heterogeneity. Acuros XB offers more accurate transport physics in such environments; therefore, future validation using Acuros XB, including subset‐based sensitivity analysis of dosimetric parameters and NTCP, may further refine the assessment of acute bowel toxicity risk and target coverage. Finally, the NTCP analysis focused on acute WB toxicity using an externally derived model, without assessment of late morbidity or prospective validation of the proposed *V*_45_‐based strata. Although our analysis was restricted to acute endpoints, BC‐driven variations in high‐dose exposure may also influence late gastrointestinal toxicity and longer‐term patient‐reported quality of life. Longitudinal follow‐up with structured patient‐reported outcome assessments in future multicenter studies, including evaluations of tumor control and late toxicity, will therefore be important to determine whether the dose response patterns identified here persist as functional or symptomatic effects over time.

## 5 CONCLUSION

Using daily iCBCT‐based dose recalculation and voxel‐wise accumulation, this study shows that inter‐fraction BC motion volume selectively increases high‐dose exposure to the PTV and WB, while near‐minimum target coverage is largely preserved. Accumulated PTV *D*_2%_ and WB hotspots (*D*_0.03cc_, *D*_150cc_) were consistently higher than planned, and larger BC expansions were associated with measurable upper‐tail dose escalations. Exploratory WB *V*_45_ bands around 300 cc and 500 cc aligned with acute toxicity strata in this retrospective cohort. These findings support prospective evaluation of accumulated WB *V*_45_ as a marker of delivered dose for on‐treatment surveillance and triage, prior to adopting any band‐based thresholds as adaptive triggers or intervention cutoffs.

## AUTHOR CONTRIBUTIONS

**Ruping Zhao**: Conception and design; manuscript writing. **Minghe Lv**: Conception and design. **Hongwei Zeng**: Conception and design; Data analysis and interpretation; Manuscript writing. **Xiangyu E**: Collection and assembly of data. **Su Zeng**: Collection and assembly of data. **Yue Feng**: Data analysis and interpretation. **Jingping Yu**: Data analysis and interpretation.

Final approval of manuscript: All authors. Accountable for all aspects of the work: All authors.

## CONFLICT OF INTEREST STATEMENT

The authors have no relevant conflicts of interest to disclose.

## ETHICS STATEMENT

This study was conducted in accordance with the Declaration of Helsinki and was approved by the Institutional Review Board of Shuguang Hospital Affiliated to Shanghai University of Traditional Chinese Medicine (Approval No. 2025‐1661‐001‐01). Written informed consent was obtained from all enrolled patients for the use of their imaging and clinical data in this research. All data were anonymized prior to analysis to ensure patient confidentiality.

## Data Availability

Data will be available on request from the authors.
